# Sleep characteristics and cognitive impairment in bipolar disorder: age-specific associations

**DOI:** 10.3389/fpsyt.2026.1804149

**Published:** 2026-03-26

**Authors:** Aidé Martínez-Cuenca, Carlota Moya-Lacasa, Alejandro García-Sánchez, Manuel Couce-Sánchez, Sergio Romero-Jiménez, Pilar Alejandra Sáiz, María Paz García-Portilla, Leticia González-Blanco

**Affiliations:** 1 Department of Psychiatry, University of Oviedo, Oviedo, Asturias, Spain; 2 Servicio de Salud del Principado de Asturias, Oviedo, Asturias, Spain; 3 Instituto de Neurociencias del Principado de Asturias, Oviedo, Asturias, Spain; 4 Instituto de Investigación Sanitaria del Principado de Asturias, Oviedo, Asturias, Spain; 5 Centro de Investigación Biomédica en Red de Salud Mental (CIBERSAM), Psychiatry, Madrid, Spain

**Keywords:** age groups, bipolar disorder, cognitive impairment, depression, sleep disturbances

## Abstract

**Introduction:**

Bipolar disorder (BD) is a chronic condition often associated with sleep disturbances and cognitive impairment, even during periods of clinical stability.

**Methods:**

This study explores the impact of insomnia on cognitive functioning in 170 outpatients diagnosed with BD, 61.8% of which were women (n=105), grouped by age (<50 years vs. ≥50 years). Standardized and validated instruments were used to assess sleep quality (Oviedo Sleep Questionnaire) and cognitive performance (Screening of Cognitive Impairment in Psychiatry).

**Results:**

Results show that 63.1% of the sample exhibited some degree of cognitive impairment, with notable differences across age groups. Although no significant differences in insomnia prevalence were found by cognitive status, lower sleep satisfaction was associated with cognitive functioning in younger patients (p=0.044). In older patients, only severity of depression was associated with cognitive impairment (p=0.049).

**Conclusions:**

These findings underscore the relevance of age-specific intervention strategies to approach cognitive impairment, focusing on sleep assessment in younger patients with BD, and rather on affective symptoms in older adults with BD.

## Introduction

Bipolar disorder (BD) is a severe chronic and episodic illness affecting more than 1% of the population worldwide. This disorder is characterized by both a high prevalence of comorbidities, significantly impacting daily functioning and quality of life, and premature mortality (1).

This disorder is characterized by mood fluctuations alternating between depressive and manic, hypomanic, or mixed episodes and stable phases of euthymia; a longer duration of this phase leads to a better quality of life (2).

Insomnia is a common and clinically relevant symptom in BD, with prevalence rates exceeding 40% in clinical samples (3) Moreover, prodromal insomnia has been reported in up to 75.6% of patients prior to depressive episodes, highlighting its potential as an early indicator of relapse (4).

There is also evidence showing that sleep disturbances can appear in all phases of the illness and even persist during euthymic phases in about 70% of patients. When considering the pattern of sleep, it is primarily associated with the nature of the episode experienced. Depressive episodes are typically characterized by an increase in nighttime awakenings and the presence of severe insomnia or hypersomnia. Conversely, manic episodes are often linked to a decreased need for sleep (5). Along with these lines, sleep anomalies have been recognized as strong predictors of developing BD, future relapse episodes (6–8) and also contribute to poorer functionality and quality of life, independent of mood state (9), as well as heightened clinical severity and suicidal ideation (1, 6).

Cognitive alterations are observed in approximately 70% of patients with BD (8, 10). Cognitive impairment has been noted to persist during the euthymic phase, impacting information processing, attention, tasks involving inhibition and long-term memory (8, 11, 12) and severely affecting executive functions even in the absence of psychotic or affective symptoms (13).

Executive functions—such as inhibition, working memory, and cognitive flexibility—have been the primary focus of investigation. Significant deficits have also been observed in areas related to verbal and non-verbal memory (10). Difficulties in language (14) and a reduction in psychomotor speed (12) are also evident. All of these features, collectively, affect learning capacity (11, 15).

A significant relationship has been established between cognitive impairment and sleep disturbances in mood disorders such mayor depresive disorder and BD (12, 16). Specifically, individuals with BD who experience sleep disturbances exhibit worse cognitive performance, even during periods of euthymia (9). Notably, the presence of both insomnia and hypersomnia has been linked to diminished performance in processing speed and inhibition (11).

Despite the evidence of significant relationship between subjective sleep disturbances and cognitive impairments in BD patients (9, 11, 12), there is an important lack of knowledge about the interplay between both of these areas. Only one study (17) adresses that improved sleep is a significant predictor of cognitive enhancement in patients with BD. Besides, no studies have analyzed differences across these areas based on age group.

This paper aims to investigate the relationship between insomnia on the cognitive functioning of patients with BD in an outpatient setting. Secondarily this study sought to investigate the association of sleep characteristics and the degree of cognitive impairment in these patients segmented by age group.

## Materials and methods

### Subjects

This study is a secondary analysis of a cross-sectional sample of 170 patients from a larger multicenter project (Ref. PI11/02493). These patients were recruited at two sites in Oviedo, Spain.

Inclusion criteria were: 1. A confirmed BD diagnosis using the Structured Clinical Interview for DSM-IV-TR Axis I Disorders (SCID-I); 2. Age >17 years; and 3. providing written informed consent to participate. The only exclusion criterion was refusal to participate in the study.

### Clinical assessment

Sociodemographic and clinical variables were collected as well as comorbidities, substance use, and psychopharmacological treatment.

To evaluate the current clinical state, validated Spanish versions of the Young Mania Rating Scale (YMRS) (18), the Hamilton Depression Rating Scale (HDRS) (19), and the Clinical Global Impression Scale (CGI-BP) (20) were used. All evaluations were performed by qualified, specialized psychologist or psychiatrist.

Euthymia was established when scores of YMRS was ≤ 6 and HDRS ≤ 7. Additionally, information on the consumption of caffeine, tobacco, alcohol, and cannabis was collected through participants’ direct testimony. A caffeine consumer was defined as anyone who reported ingesting at least one cup of caffeinated coffee per day. Similarly, a tobacco consumer was defined as an individual who reported smoking at least one cigarette daily, an alcohol consumer as someone who reported drinking at least one standard drink per week, and a cannabis user as someone who reported having consumed it in the last month.

### Sleep characteristics

Sleep quality in the past month was evaluated using the Oviedo Sleep Questionnaire (OSQ) (21). The OSQ is a brief, semi-structured interview consisting of 15 items, 10 of which provide scores on two dimensions: subjective sleep satisfaction (1 item) and severity of insomnia (9 items). Each OSQ item was considered a continuous variable from lesser to greater severity [1 to 5], except for item 1 (Sleep Satisfaction) [1 to 7], which goes from very dissatisfied to very satisfied. This instrument has been validated in populations with severe mental disorders, such as schizophrenia and BD (22).

### Cognitive performance

To assess cognitive performance, the Spanish version of the Screen for Cognitive Impairment in Psychiatry (SCIP) was used. This is a brief, validated test for measuring deficits in various cognitive domains. It consists of five subtests that evaluate different cognitive domains: Verbal Learning; Working Memory; Verbal Fluency; Processing Speed; and Delayed Memory. Cognitive impairment was defined as a dichotomous variable (‘impaired’ vs. ‘unimpaired’) for each cognitive domain separately. The classification was based on the optimal, empirically validated cut-off scores proposed by Rojo et al. (23) in the validation study of the SCIP. The severity levels can be normal (0 to 2 affected domains), mild (3 affected domains), moderate (4 affected domains), or severe (5 affected domains). In order to provide the necessary statistical power to conduct meaningful age-stratified multivariate analyses, the SCIP score was recoded into a categorical variable: no or mild cognitive impairment, and moderate to severe cognitive impairment.

### Statistical analysis

Data analysis was performed using IBM SPSS Statistics 23.0 for Windows software package. The statistical significance level was set at a 95% confidence level (α). The significance level was set at p < 0.05. Frequencies, percentages and mean and standard deviation were used to describe the characteristics of the sample. Because of the physiological changes associated to aging (regarding sleep, cognitive performance and menopause) (24, 25), as well as clinically relevant transitions around this age towards late-life BD (26), the sample was divided into two groups to carry out the statistical analysis: <50 years old and ≥ 50 years. This cut-off point may also reflect differences in illness burden, particularly cognitive performance. To detect differences between groups, Chi-squared (χ²) test and T-test (t) were used. A multivariate logistic regression with SCIP score categorized in no-to-mild cognitive impairment and moderate-to-severe impairment as a dependent variable to adjust for covariates.

### Ethical considerations

The Clinical Research Ethics Committee of Hospital Universitario Central de Asturias in Oviedo approved the study protocol (refs. 36/12). Written informed consent was obtained from all participants prior to enrolment.

## Results

### Sample characteristics

The total sample included 170 participants, 61.8% of which were women (n=105), with a mean age of 49.19 (SD = 12.88). The sample was segmented in two groups depending on age: < 50 years old (n=91; 53.5%) and ≥ 50 years (n=79; 46.5%).

BD type I was diagnosed in 61.2% of the sample. Out of the total sample, 76 patients (44.7%) were in euthymia, while 74 (43.5%) met criteria for depression based on their HDRS score. A minority group (11.8%) presented with criteria for hypomania/mania or a mixed episode. Detailed demographic and clinical data for the total sample and for the two age groups are shown in **Table 1**.

**Table 1:** Demograpfic and clinical characteristics of the total sample.

Demographics	Total sample (n=170)	< 50 years (n=91; 53.5%)	≥ 50 years (n=79; 46.5%)	Statistic	P-value
Age (mean (SD))	49.19 (12.88)	39.54 (7.31)	60.30 (7.99)	17.570¹	**0.001**
Sex, female (n (%))	105 (61.80)	56 (61.50)	49 (62.00)	0.004²	0.950
Marital Status, married/partner (n (%))	72 (42.40)	31 (34.10)	41 (51.90)	5.510²	**0.020**
Active Employment (n (%))	31 (18.20)	18 (19.80)	13 (16.50)	0.310²	0.580
Years of education (mean (SD))	13.46 (5.24)	13.98 (5.02)	12.86 (5.46)	1.360¹	0.175
Type, BD I (n (%))	104 (61.20)	63 (69.20)	41 (51.90)	5.350²	**0.020**
Age of Onset (mean (SD))	27.03 (10.92)	23.30 (7.62)	31.36 (12.52)	-5.055¹	**0.001**
Age of Diagnosis (mean (SD))	36.79 (12.90)	31.30 (8.00)	42.97 (14.51)	-6.356¹	**0.001**
N° of Hospitalizations (mean (SD))	2.35 (3.12)	2.43 (3.01)	2.26 (3.25)	0.364¹	0.717
HDRS Depression (mean (SD))	9.32 (7.21)	10.46 (7.57)	8.00 (6.57)	2.270¹	**0.025**
YMRS Mania (mean (SD))	3.62 (4.59)	4.01 (4.56)	3.16 (4.61)	1.200¹	0.232
CGI (mean (SD))	4.08 (1.16)	4.22 (1.17)	3.94 (1.11)	1.565¹	0.120

¹ Refers to the t-statistic for a t-test. ² Refers to the chi-square statistic.

SD, standard deviation; BD, bipolar disorder; HDRS, Hamilton Depression Rating Scale; YMRS, Young Mania Rating Scale; CGI, Clinical Global Impression.

P-values in bold: statistically significant differences.

Out of the total sample, 75.3% (n=128) were on conventional mood stabilizers (lithium, valproate, lamotrigine, oxcarbazepine), lithium being the most frequently used (41.8%, n=71). Almost the entire sample was on antipsychotics (98.8%, n=168). Antidepressants were included in the treatment of 46.5% patients (n=79) and more than half of the patients took benzodiazepines (59.4%, n=101). No significant differences were observed between age groups (p>0.05) for any of the psychopharmacological treatments.

Of the 170 patients, 99 (60%) consumed caffeinated coffee, with an average daily consumption of 2.21 ± 1.52 cups. Daily smoking was reported by 43.4% of the participants, with an average of 16.04 ± 10.79 cigarettes per day. A total of 66 patients (26.1%) acknowledged weekly alcohol consumption, with an average intake of 5.10 ± 5.73 standard alcohol units per week. Finally, only 7 patients (4.2%) had consumed cannabis at least once in the last month. No significant differences were observed between the age groups (p>0.05).

### Cognitive impairment

From the total sample, 63.1% showed some degree of global cognitive impairment (at least 3 domains out of 5 were affected). Regarding severity of cognitive impairment, 14.9% presented severe impairment, 23.8% moderate impairment, and 24.4% mild impairment. In terms of specific cognitive domains, visuomotor tracking was impaired in 76.8% of the sample, followed by verbal learning in 70.4%. Delayed recall was impaired in 59.8% of participants, while working memory reached 58.6%. Finally, verbal fluency had the lowest rate of impairment at 26.2%.

In the group of participants under 50 years old, 51.6% had some degree of impairment compared to 76.6% in the ≥ 50 age group (χ²=11.173; p<0.001). Statistically significant differences were observed in the severity of cognitive impairment (χ²=7.963; p=0.019). In the <50 age group, 5.5% of participants had severe cognitive impairment, 22% moderate, and 24.2% mild. In contrast, among those ≥ 50, 26% had severe cognitive impairment, 26% moderate, and 24.7% mild.

To maximize statistical power given the sample size, cognitive impairment severity was grouped into two levels: no-to-mild vs moderate-to-severe. Higher mean age was observed in participants with moderate-to-severe cognitive impairment compared to those with mild or without impairment (54.7 ± 14.5years vs 45.5 ± 10.4 years, p<0.001).

### Sleep characteristics

Out of the total sample, 44 individuals (25.9%) met ICD-10 criteria for insomnia. Contrary to what was expected, prevalence of insomnia was higher in the younger age group (31.9% vs 19.0%) showing a trend towards statistical significance (p=0.05). However, severity of insomnia did not differ between both groups (18.40 ± 8.45 vs 18.36 ± 10.86 years, p>0.05).

In relation to sleep satisfaction, in the <50 group, 53.9% were satisfied vs 60.8% in the ≥ 50 (p>0.05).

Sleep quality, assessed using the OSQ, is presented in **Supplementary Material** (**Supplementary Table 1**). The under-50 group reported greater difficulties due to sleepiness (2.54 ± 1.66 vs 1.90 ± 1.46, p=0.009), higher frequency of daytime sleepiness (2.36 ± 1.60 vs 1.86 ± 1.40, p=0.035) and poorer daytime performance due to sleepiness (2.33 ± 1.60 vs 1.71 ± 1.38, p=0.011).

Regarding sex differences across the entire sample, a statistically significant difference was only identified for excessive sleepiness, which was higher in women than in men (2.42 ± 1.65 vs 1.92 ± 1.45, p=0.042).

### Relationship between sleep and cognitive performance

In the under-50 age group, no significant differences were found in the prevalence of insomnia based on the degree of cognitive impairment (χ²=0.860; p=0.354).

Although the prevalence of insomnia in those aged 50 and older was twice as high in patients without or with mild impairment compared to those with moderate to severe impairment, the difference did not reach statistical significance (25% vs. 12,2%; χ²=2.113; p=0.146). Furthermore, no significant differences were found in the prevalence of insomnia based on which specific cognitive domains were affected (p>0.05).

While there were no differences in the prevalence of insomnia based on the degree of cognitive impairment, some differences were observed in sleep characteristics, with distinct findings for each age group. Participants under 50 with moderate-to-severe impairment reported being less satisfied with sleep than those without or with mild impairment (3.44 ± 2.04 vs 4.42 ± 1.85),a statistically significant difference (p=0.044). In the group aged 50 and older, those with moderate-to-severe impairment showed greater difficulty staying asleep (2.50 ± 1.80 vs 1.73 ± 1.44, p=0.043). This group of patients also reported greater difficulty waking up at their usual time (2.25 ± 1.68 vs 1.62 ± 1.16), with a trend towards significance (p=0.062). No other associations were observed between cognitive impairment and sleep characteristics (**Table 2**).

**Table 2:** Relationship between sleep and cognitive functioning.

Sleep characteristics	< 50 years (n=91)			≥ 50 years (n=77)		
	No or mild impairment (n=66)	Moderate–severe impairment (n=25)	T-test (p)	No or mild impairment (n=36)	Moderate–severe impairment (n=41)	T-test (p)
Sleep satisfaction	4.42 (1.85)	3.44 (2.04)	2.081 (**0.044**)	4.65 (1.93)	4.45 (2.03)	0.440 (0.661)
Initial insomnia	2.19 (1.62)	2.38 (1.71)	-0.464 (0.646)	1.92 (1.48)	1.90 (1.56)	0.055 (0.957)
Middle insomnia	2.35 (1.63)	2.39 (1.72)	-0.091 (0.928)	1.73 (1.41)	2.50 (1.80)	-2.061 (**0.043**)
Poor restorative sleep	2.39 (1.74)	2.38 (1.78)	0.038 (0.970)	1.78 (1.41)	2.17 (1.69)	1.095 (0.277)
Late insomnia	2.17 (1.62)	2.26 (1.57)	-0.231 (0.819)	1.62 (1.16)	2.25 (1.68)	-1.897 (**0.062**)
Excessive sleepiness	2.59 (1.64)	2.39 (1.75)	0.483 (0.632)	1.68 (1.25)	2.10 (1.63)	-1.275 (0.206)
Sleep onset latency	2.17 (1.35)	2.36 (1.60)	-0.527 (0.601)	2.35 (1.51)	1.95 (1.43)	1.196 (0.237)
Night awakenings	2.51 (1.35)	2.48 (1.41)	0.084 (0.933)	2.27 (1.38)	2.62 (1.51)	-1.036 (0.303)
Early awakening	1.87 (1.43)	2.16 (1.52)	-0.812 (0.421)	2.00 (1.56)	1.78 (1.36)	0.670 (0.505)
Sleep efficiency	1.85 (1.26)	2.00 (1.35)	-0.485 (0.631)	1.78 (1.22)	1.85 (1.47)	-0.200 (0.842)
Work/social impact due to fatigue	2.35 (1.55)	2.50 (1.64)	-0.638 (0.708)	1.84 (1.30)	1.64 (1.34)	0.648 (0.519)
Daytime sleepiness	2.37 (1.57)	2.32 (1.67)	-0.127 (0.900)	1.75 (1.29)	1.95 (1.52)	0.619 (0.538)
Social impact due to sleepiness	2.31 (1.57)	2.38 (1.71)	-0.156 (0.877)	1.67 (1.24)	1.73 (1.45)	-0.189 (0.851)
Snoring	3.06 (1.91)	2.48 (1.90)	1.260 (0.215)	3.38 (1.87)	3.28 (1.90)	0.222 (0.825)
Snoring with suffocation	1.52 (1.29)	1.35 (0.98)	0.670 (0.506)	1.81 (1.54)	1.61 (1.49)	0.566 (0.573)
Restless legs	2.11 (1.61)	1.63 (1.27)	1.329 (0.187)	1.86 (1.61)	1.77 (1.61)	0.234 (0.816)
Nightmares	2.20 (1.45)	1.88 (1.29)	1.016 (0.315)	1.80 (1.41)	2.13 (1.49)	-0.973 (0.334)
Sleep aids	3.44 (1.89)	4.00 (1.68)	-1.281 (0.204)	3.33 (2.00)	3.80 (1.80)	-1.071 (0.288)

P-values in bold: statistically significant differences.

A multivariate logistic regression analysis was performed to adjust for covariates. The dependent variable was the level of cognitive impairment based on SCIP scores, categorized as moderate-to-severe versus no to mild impairment. In the younger group, the association of sleep satisfaction with severity of cognitive impairment remained significant after controlling for clinical and pharmacological covariates (OR = 0.73; 95% CI: 0.56–0.96; p=0.024), indicating that higher perceived sleep satisfaction was associated with a lower likelihood of cognitive impairment. However, the variables such as; use of antidepressants, antipsychotics, mood stabilizers other than lithium, current lithium treatment, benzodiazepines, global clinical severity, total depression (HDRS) and mania (YMRS) scores, sex, and daily caffeine intake, did not enter the model (**Table 3**). In the older group the regression model included middle insomnia (OSQ 2.2) and late insomnia (OSQ 2.4*)*, together with the same covariates listed above. However, depression severity (HDRS) was the only variable that entered the model (OR = 1.086; 95% CI: 1.000–1.179; p=0.049) (**Table 3**).

**Table 3:** Logistic regression analysis for cognitive impairment.

Participants < 50 years							
Variable	B	SE	Wald	P	OR (ExpB)	95% CI for OR (Lower–Upper)	Entered in model
OSQ1 (Sleep satisfaction)	−0.315	0.139	5.12	0.024	0.73	0.555–0.959	✓
Antidepressants	—	—	—	0.626	—	—	×
Antipsychotics	—	—	—	0.321	—	—	×
Other mood stabilizers	—	—	—	0.192	—	—	×
Lithium treatment	—	—	—	0.688	—	—	×
Benzodiazepines	—	—	—	0.238	—	—	×
Clinical severity	—	—	—	0.583	—	—	×
HDRS	—	—	—	0.251	—	—	×
YMRS	—	—	—	0.136	—	—	×
Sex (Female)	—	—	—	0.434	—	—	×
Caffeine/day	—	—	—	0.291	—	—	×
Participants ≥50 years							
HDRS	0.082	0.042	3.88	0.049	1.09	1.00–1.179	✓
OSQ 2.2 Middle insomnia	—	—	—	0.430	—	—	×
OSQ 2.4 Late insomnia	—	—	—	0.184	—	—	×
Antidepressants	—	—	—	0.821	—	—	×
Antipsychotics	—	—	—	0.348	—	—	×
Other mood stabilizers	—	—	—	0.555	—	—	×
Lithium treatment	—	—	—	0.465	—	—	×
Benzodiazepines	—	—	—	0.418	—	—	×
Clinical severity	—	—	—	0.931	—	—	×
YMRS	—	—	—	0.993	—	—	×
Sex (female)	—	—	—	0.072	—	—	×
Caffeine/day	—	—	—	0.716	—	—	×

Model statistics for participants <50 years: Cox & Snell R² = 0.075; Nagelkerke R² = 0.104; Model statistics for participants ≥50 years: Cox & Snell R² = 0.063; Nagelkerke R² = 0.085.

CI not computed for variables excluded from the final model.

OSQ, Oviedo Sleep Questionnaire; HDRS, Hamilton Depression Rating Scale, YMRS, Young Mania Rating Scale.

P-values in bold: statistically significant differences.

## Discussion

The findings of this study provide valuable insights into the complex interaction between sleep disturbances and cognitive functioning in patients with bipolar disorder (BD), while also considering the modulating effect of age. Although sleep disturbances are well documented in this population, our results nuance this evidence, suggesting that not all sleep characteristics have the same impact on cognition, and that this relationship varies according to age group.

Prevalence of insomnia (25.9%) was slightly lower than previously reported rates, which range from 40% to 75.6% depending on the assessment instrument and clinical phase (3–5, 12, 27). Even though younger patients had a higher prevalence of insomnia, these differences were not statistically significant, in line with published literature (3, 16). There is a scarcity of scientific evidence directly addressing the relationship between age and sleep quality in patients with bipolar disorder. Nevertheless, a cross-sectional study conducted in Norway (3) reveals that insomnia is more frequent in earlier age of onset of BD and suggests that sleep disturbances may be more closely linked to the chronicity of bipolar disorder rather than to chronological age per se. However, age might be moderating the consequences of poor sleep quality. In our study, younger patients reported greater excessive sleepiness and associated social impact, as well as higher socio-occupational impact due to fatigue. This could be related to the fact that younger patients with BD have better socio-ocupational functioning and face higher social and professional demands, amplifying the perceived impact of sleep disturbances compared to older patients.

Regarding cognitive performance, over 60% of participants exhibited some degree of impairment, similarly to previous results (28). More severe deficits were observed in older individuals, consistently with previous studies (29). The most affected domains were visuomotor tracking and verbal learning, which aligns with prior findings identifying these areas as particularly vulnerable in BD (30).

Concerning the interaction between sleep disturbances and cognitive impairment, no significant associations were found between insomnia prevalence and the degree of cognitive impairment in either age group. However, certain sleep characteristics were linked to cognitive performance in younger patients, with lower sleep satisfaction correlated with poorer cognitive functioning. In contrast, although older individuals with greater cognitive impairment showed more difficulties maintaining sleep, this association did not persist after controlling for covariates. Instead, only the severity of depressive symptoms was found to be associated with cognitive dysfunction in this age group.

These findings highlight a potential pivotal role of sleep quality in the cognitive function of younger BD patients, even more important than other clinical features. Moreover, this relationship was not seen to be bidirectional, which means that poorer sleep quality could predict worse cognitive performance, but not the other way around (16). Conversely, these age-related differences may reflect developmental and degenerative processes. In younger individuals, an underdeveloped prefrontal cortex and immature top-down control mechanisms could heighten vulnerability to the emotional and cognitive effects of poor sleep (31). Additionally, greater functional and social demands in this age group may amplify the subjective impact of sleep disturbances, increasing self-awareness of sleep-related dysfunction (32).

In older patients, however, cognitive impairment is associated with the severity of depressive symptoms, consistent with previous literature (33) rather than with sleep disturbances. This is particularly relevant for older patients experiencing high-burden depressive episodes (34, 35), and not during remission (36, 37). Specifically in BD, cognitive deficits observed during depressive episodes appear to be state-related and comparable to those reported in unipolar depression, suggesting that current affective symptoms, rather than diagnosis, play a central role in cognitive impairment (34, 37). These findings indicate that later in life, the contribution of sleep quality to cognitive impairment could be overshadowed by depression. This suggests that modifiable behavioral and physiological factors may have a greater impact on cognition earlier in the course of BD, before the cumulative effects of age and affective symptom burden become more prominent.

Clinically, these findings highlight the need for comprehensive sleep assessments in patients with BD, even during stable phases, focusing not only on the presence of insomnia but also on subjective sleep quality and daytime functioning, specifically in younger patients. Moreover, the results advocate for age-specific intervention strategies in order to improve cognitive dysfunction, prioritizing emotional well-being and sleep perception in younger adults, and affective symptoms in older patients.

This study has limitations. Its cross-sectional design limits causal inference, and the absence of objective sleep measures restricts generalizability. Because most participants were euthymic or depressed, larger manic/hypomanic samples are needed to analyze the possible contribution of mania to cognitive impairment. Moreover, further research should include sex-stratified analysis given that specific hormonal changes in women have been shown to affect sleep architecture and cognitive performance (38). Despite the limited explanatory power of the regression model due to the low explained variance, the observed associations highlight relevant clinical targets for future research and clinical assessment.

Among the strengths of this study are the adequate sample size, which allowed stratified subgroup analyses, and the use of non-invasive, validated subjective tools for both sleep and cognition assessment, collecting clinically relevant information, making the study easier to replicate, more feasible for patients to participate in all stages of the disorder, and allowing larger samples to be studied as an initial step to guide future research using objective methods.

Our findings emphasize the necessity of assessing sleep quality within the full context of the patient’s clinical presentation. The interplay between sleep and cognition is complex and age-dependent, suggesting that standard treatments may need to be adapted. These insights support the move towards more comprehensive care models in BD that simultaneously target sleep disturbances and cognitive preservation.

## Conclusion

Age modulates the interaction between sleep and cognitive performance, such that this interaction is observed exclusively in individuals under 50 years of age. In this age group, lower sleep satisfaction is associated with poorer cognitive performance. Conversely, in those ≥ 50, cognitive decline shows modest association with the severity of depressive symptoms. These findings reinforce the need for an in-depth assessment of sleep disorders and underscore the potential benefit of age-tailored interventions: prioritizing sleep management in younger patients, while emphasizing the control of depressive symptoms in older adults. Such a personalized approach could enhance early detection and comprehensive management, yielding potential clinical and functional benefits.

## Data Availability

The raw data supporting the conclusions of this article will be made available by the authors, without undue reservation.
