# Correction: Sleep characteristics and cognitive impairment in bipolar disorder: age-specific associations

**DOI:** 10.3389/fpsyt.2026.1840713

**Published:** 2026-03-31

**Authors:** Frontiers Production Office

**Affiliations:** Frontiers Media SA, Lausanne, Switzerland

**Keywords:** age groups, bipolar disorder, cognitive impairment, depression, sleep disturbances

Affiliation “Servicio de Salud del Principado de Asturias, Oviedo, Asturias, Spain” was erroneously given as “Psychiatry department, Servicio de Salud del Principado de Asturias, Oviedo, Asturias, Spain”.

Affiliation “Instituto de Neurociencias del Principado de Asturias, Oviedo, Asturias, Spain” was erroneously given as “Psychiatry department, Facultad de Psicología, Instituto de Neurociencias del Principado de Asturias, Oviedo, Asturias, Spain”.

Affiliation “Instituto de Investigación Sanitaria del Principado de Asturias, Oviedo, Asturias, Spain” was erroneously given as “Psychiatry department, Instituto de Investigación Sanitaria del Principado de Asturias, Oviedo, Asturias, Spain”.

The funder “the Government of the Principality of Asturias (Ref IDE/2024/774)” erroneously omitted.

The original version of this article has been updated.

