# Correction: Sleep characteristics and cognitive impairment in bipolar disorder: age-specific associations

**DOI:** 10.3389/fpsyt.2026.1898562

**Published:** 2026-06-18

**Authors:** Aidé Martínez-Cuenca, Carlota Moya-Lacasa, Alejandro García-Sánchez, Manuel Couce-Sánchez, Sergio Romero-Jiménez, Pilar Alejandra Sáiz, María Paz García-Portilla, Leticia González-Blanco

**Affiliations:** 1 Department of Psychiatry, University of Oviedo, Oviedo, Asturias, Spain; 2 Servicio de Salud del Principado de Asturias, Oviedo, Asturias, Spain; 3 Instituto de Neurociencias del Principado de Asturias, Oviedo, Asturias, Spain; 4 Instituto de Investigación Sanitaria del Principado de Asturias, Oviedo, Asturias, Spain; 5 Centro de Investigación Biomédica en Red de Salud Mental (CIBERSAM), Psychiatry, Madrid, Spain

**Keywords:** age groups, bipolar disorder, cognitive impairment, depression, sleep disturbances

Complete details for the funder “Government of the Principality of Asturias” were missing. The correct **Funding** statement is:

“The author(s) declared that financial support was received for this work and/or its publication. This work was carried out thanks to the Carlos III Healthcare Institute, the Spanish Ministry of Science, Innovation and Universities, the European Regional Development Fund (ERDF/FEDER) (PI11/02493). This project is also funded by the Government of the Principality of Asturias through the Agency for Science, Business Competitiveness and Innovation of the Principality of Asturias, and co-funded by the European Union through the Grants for Research Groups of Organisations of the Principality of Asturias for the 2024 financial year, under file number IDE/2024/00774. The sponsors had no further role in the study design, collection analysis and interpretation of data, writing of the report, or the decision to submit this paper for publication.”

The original version of this article has been updated.

