# Supplementary material for: Sleep characteristics and cognitive impairment in bipolar disorder: age-specific associations
Source: Front Psychiatry. 2026 Mar 26;17:1804149. doi: 10.3389/fpsyt.2026.1804149 (PMC13077464; doi:10.3389/fpsyt.2026.1804149)
Supplement: Supplementary file 1 [file Table1.docx]

**Supplementary Materials**

**Table S1.** Sleep Characteristics of the total sample and segmented by age.

| **Sleep Characteristics (mean (SD))** | **Total Sample (n=170)** | **< 50 years (n=91)** | **≥ 50 years (n=79)** | **t-test** | **p-value** |
| --- | --- | --- | --- | --- | --- |
| **Sleep Satisfaction** | 4.32 (1.97) | 4.14 (1.95) | 4.52 (1.99) | 1.253 | 0.211 |
| **Initial Insomnia** | 2.10 (1.60) | 2.24 (1.64) | 1.94 (1.54) | 1.227 | 0.222 |
| **Middle Insomnia** | 2.26 (1.66) | 2.36 (1.65) | 2.14 (1.67) | 0.872 | 0.384 |
| **Poor Restorative Sleep** | 2.21 (1.66) | 2.39 (1.72) | 2.01 (1.59) | 1.460 | 0.146 |
| **Late Insomnia** | 2.09 (1.56) | 2.20 (1.60) | 1.97 (1.50) | 0.915 | 0.362 |
| **Excessive Sleepiness** | 2.23 (1.60) | 2.54 (1.66) | 1.90 (1.50) | 2.634 | **0.009** |
| **Time to Fall Asleep** | 2.20 (1.45) | 2.22 (1.42) | 2.16 (1.50) | 0.256 | 0.798 |
| **Awakenings During Sleep** | 2.47 (1.40) | 2.50 (1.37) | 2.44 (1.45) | 0.294 | 0.769 |
| **Early Awakening** | 1.93 (1.47) | 1.95 (1.45) | 1.91 (1.49) | 0.189 | 0.850 |
| **Sleep Efficiency** | 1.87 (1.33) | 1.89 (1.28) | 1.85 (1.39) | -0.201 | 0.841 |
| **Socio-occupational Impact due to Fatigue** | 2.10 (1.50) | 2.39 (1.57) | 1.77 (1.36) | 2.727 | **0.007** |
| **Daytime Sleepiness** | 2.13 (1.53) | 2.36 (1.60) | 1.86 (1.40) | 2.127 | **0.035** |
| **Social Impact due to Sleepiness** | 2.05 (1.53) | 2.33 (1.60) | 1.71 (1.38) | 2.561 | **0.011** |
| **Snoring without Apnea** | 3.10 (1.91) | 2.91 (1.92) | 3.32 (1.88) | -1.392 | 0.166 |
| **Snoring with Apnea** | 1.57 (1.36) | 1.48 (1.22) | 1.68 (1.50) | -0.968 | 0.335 |
| **Restless Legs** | 1.89 (1.56) | 1.98 (1.53) | 1.79 (1.59) | 0.762 | 0.447 |
| **Nightmares** | 2.04 (1.42) | 2.11 (1.40) | 1.95 (1.40) | 0.740 | 0.460 |
| **Sleep Aids** | 3.61 (1.86) | 3.60 (1.84) | 3.62 (1.88) | 0.915 | 0.362 |

SD (±): Standard Deviation

## 
